# The Reliability and Validity of the Telephone-Based and Online Polish eHealth Literacy Scale Based on Two Nationally Representative Samples

**DOI:** 10.3390/ijerph16173216

**Published:** 2019-09-03

**Authors:** Mariusz Duplaga, Karolina Sobecka, Sylwia Wójcik

**Affiliations:** 1 Department of Health Promotion, Institute of Public Health, Faculty of Health Sciences, Jagiellonian University Medical College, 31-531 Krakow, Poland; 2 Scientific Student’s Circle of Health Promotion, Department of Health Promotion, Institute of Public Health, Faculty of Health Sciences, Jagiellonian University Medical College, 31-531 Krakow, Poland

**Keywords:** ehealth literacy, health literacy, ehealth literacy scale, validity, reliability, survey

## Abstract

Adequate ehealth literacy is one of the key instruments safeguarding people against unreliable health-related information obtained from the Internet. This paper presents an assessment of the reliability and the validity of a Polish version of the ehealth literacy scale (Pl-eHEALS). The assessment was carried out on the basis of two nationally representative samples of the Polish population. In the first survey of adults at least 50 years old, the technique of computer-assisted telephone interviewing (CATI) was applied. In the second survey of young adult women (18–35 years old), the technique of computer-assisted web interviewing (CAWI) was used. The reliability and the validity of the Pl-eHEALS was analyzed. There were no floor or ceiling effects revealed in either sample. The Cronbach’s alpha coefficients were 0.90 and 0.88, and Guttman split-half coefficients were 0.89 and 0.81, respectively. Exploratory factors analysis revealed single factor models in both cases. The sum of squared loadings in the first survey was 6.090 and accounted for 58.72% of the variance. In the second survey, the sum was 5.927 and was responsible for 55.06% of the variance. Hypothesis testing showed that, for older adults, higher ehealth literacy was prevalent in the respondents who used the Internet more frequently. Among young adult women, higher readiness to use the Internet as a primary source of health-related information and to undertake specific internet health-related activities was associated with higher ehealth literacy. The analysis reported in this paper confirmed the reliability and the validity of the instrument. It should be stressed that, prior to this study, there was no validated Polish version of the eHEALS that could be used with Polish-speaking respondents.

## 1. Introduction

Patient empowerment is one of the key objectives for modern health care systems [1]. It requires a new perspective to be defined regarding the role of the patient in interaction with health care providers. All citizens and patients are expected to take responsibility for their health, to actively engage in self-care activities and, if possible, to became partners—instead of passive recipients—of communication coming from health care professionals [2]. 

This change in the perception of a patient’s role has been mediated to a great extent by the growing availability of health information on the Internet [3,4,5]. However, the use of the Internet for accessing health information may be perceived both as an opportunity and a challenge. The problem with the reliability of health information available online was recognized from the beginning of the Internet revolution. One of the approaches combating the potential risks resulting from accessing unreliable health information on the Internet was based on the certification or even the automatic verification of such resources [6]. The well-known Health on the Net Foundation established in the mid-1990s is an example of such an initiative [7]. Unfortunately, accreditation and certification actions quickly became inadequate because of the intense growth of Internet resources. In 2002, Gagliard and Jadad identified about 50 such initiatives [8]. It seems that an approach relying on enhancing the competencies of Internet users may be more efficient. The provision of adequate and appropriate education and training for society could prepare people to safely handle health-related Internet-based information. As early as 2002, Purcell et al. indicated that properly preparing the authors of Internet content could be one of the possible solutions [9]. In 2006, Norman and Skinner introduced the concept of ehealth literacy relying on six fundamental literacies: traditional, health, information, scientific, media, and computer (Lily model) [10]. The definition of ehealth literacy proposed by these authors stemmed from that proposed by the Institute of Medicine two years earlier [11]. According to the authors, ehealth literacy is the set of abilities including searching for, finding, understanding, and appraising the health information available from electronic sources and the ability to apply the knowledge gained to solve health related problems [10]. 

Norman and Skinner developed and validated a tool, eHealth Literacy Scale (eHEALS), enabling the assessment of Internet users’ ehealth literacy [12]. The tool consists of eight items asking about the perception of a person’s own skills related to searching, finding, evaluating, and applying health information found on the Internet. The responses to the items included in eHEALS are based on the 5-point Likert scale ranging from strongly disagree to strongly agree with a neutral option in the middle. The total score that can be achieved after responding to eHEALS surveys ranges from 8–40. Initially, the eHEALS scale was validated on a group of 664 school students of mean age 14.95 years. Exploratory factor analysis resulted in the single factor structure with an eigenvalue of 4.48 accounting for 56% of the variance of the scale [12]. 

After several years, the theoretical justification for the eHEALS was questioned. Even Norman, in his 2011 paper, indicated that the concept of ehealth literacy from 2006 could be challenged because of phenomena such as the accelerated growth of social media and changes to the Internet users’ roles. These include both information finding and providing as well as new, innovative methods of using the electronically obtained information to solve problems [13]. Some authors indicated that eHEALS does not actually correspond to all six types of literacies included in the Lily model [14]. Others tended to question the validity of the eHEALS instrument [15]. Recently, other tools to measure ehealth literacy have been proposed, including a three-dimensional eHealth Literacy Scale (eHLS) developed by Hsu et al. [16] and a multidimensional, seven-scale ehealth literacy questionnaire developed by Kayser et al. [17]. 

Despite all the criticism, eHEALS is widely used to assess ehealth literacy. It has demonstrated good internal consistency in most studies, and its brevity adds to its popularity. It should be recognized that eHEALS has been used in paper-, telephone- and Internet-based surveys. From 2006, the tool was validated in many studies performed on diversified groups of respondents: adults [18,19,20,21,22], older adults [23,24,25,26,27,28], university students [29,30,31], and adolescents [12,32,33,34,35]. It was also applied in surveys carried out on various groups of patients: recipients of ambulatory care [36,37], patients with chronic diseases [22,38,39,40,41], patients suffering from rheumatic diseases [15], human immunodeficiency virus (HIV) positive persons [42,43], primary lung cancer survivors [44], and parents of children with special health care needs [45,46]. Currently, there are also many national versions available, including German [47], Greek [48], Yidisz [19], Japanese [18], Dutch [15], Chinese [32,40], Korean [49], Turkish [50], Persian [35,51], Portuguese [33], Italian [52,53,54], Spanish [55,56], Arabic [31], and Serbian [57].

This paper reports the results of the assessment of the validity and the reliability of the Polish version of the eHEALS. The questionnaire was developed in line with rules of transcultural adaptation and applied in two large surveys performed on nationally representative samples.

## 2. Materials and Methods 

### 2.1. Surveys

The technique of computer-assisted telephone interviewing (CATI) was applied in the survey undertaken with adults of at least 50 (50+) years old (*n* = 1000) recruited by a third party, Biostat Company (Biostat Sp. z o.o., Rybnik, Poland) [58]. A computer-assisted web-based technique was used for interviewing a representative sample of 18–35 years old Polish women (*n* = 1030). The survey was carried out by the Partner in Business Strategies (PBS) Company (PBS Sp. z o.o., Sopot, Poland) [59]. Both companies undertake various forms of opinion polls. The companies collecting the data were selected in accordance with the required tender procedures binding public organizations in Poland. The respondents selected for participation in the CATI study resulted from stratified proportional sampling from the data base of mobile and stationary phone numbers used by the Biostat Company. For the online study, the respondents were recruited from a certified Internet panel of the PBS company (quality certificate based on the Interviewer Quality Control Scheme, IQCS) [60]. The sample reflected the structure of Polish society regarding age, education, location of residence, and Nomenclature of Territorial Units for Statistics (NUTS) 1 region in a relevant group of women.

The primary goal for both surveys was an assessment of health and ehealth literacy in relevant populations and their association with variables reflecting health and information behaviors, use of health care resources, and their opinions on selected public health issues, e.g., vaccination. The secondary goal was the validation of adapted tools for the assessment of health and ehealth literacy. In this paper, the results of the validation of the Polish version of eHEALS scale are reported. 

The research activities reported in the paper received the consent of the Bioethical Committee of Jagiellonian University in Krakow (No 122.6120.313.2016 of November 24, 2016).

### 2.2. Questionnaires

The questionnaires used in both surveys consisted of a short, 16-item version of the European Health Literacy Survey questionnaire (HLS-EU-Q16) [61]; an 8-item Polish version of the eHEALS tool (Pl eHEALS); a set of questions exploring health behaviors and the use of Internet-based resources in the context of health; and questions exploring the socio-demographic and economic profile of the respondents. The items included in the questionnaires employed in both surveys differed in respect to the items related to health behaviors and Internet activities. The questionnaire for the survey designed for respondents aged 50+ consisted of 54 items and that for the sample of women in the 18–35 age group consisted of 57 items.

### 2.3. Pl-eHEALS 

The Polish version of eHEALS (Pl-eHEALS) was prepared according to the guidelines of transcultural adaptation recommended by the World Health Organization [62] but with some modifications of the process, as described below. Permission to use the tool was given by Dr. Cameron D. Norman, one of the authors of the eHEALS instrument. The forward translation of the eHEALS was conducted by a professional with Polish as a mother tongue with a broad background in medicine and public health, extensive experience in ehealth and telemedicine, and advanced English language skills. The translated version was discussed and accepted by an expert panel consisting of five persons—three researchers from the Department of Health Promotion and two students active in the Scientific Student’s Circle of Health Promotion. A back-translation was carried out by an independent bilingual translator without knowledge of the initial questionnaire. After explaining the differences between the original and the back-translated versions of the questionnaire, it was pretested on a group of 10 public health students. In this group, cognitive interviewing was also performed. The understanding of items included in the questionnaire was also discussed with four older subjects identified by the members of the expert panel during face-to-face individual meetings. The structure of the questionnaire was discussed in a focus meeting, and the final version was agreed upon.

### 2.4. Data Analysis

Statistical analysis was carried out using the software package, IBM SPSS v.24 (IBM Corp, Armonk, NY, USA). Descriptive statistics were provided for sociodemographic variables [absolute and relative frequency, means, and standard deviations (SD)]. 

### 2.5. Reliability

Floor effects and ceiling effects were assessed on the basis of the percentage of participants who scored 8 or 40, respectively. Internal consistency of the Pl-eHEALS instrument was analyzed with the Cronbach’s alpha coefficient. As a test-retest analysis could not be performed with the available survey designs, the Guttman split-half coefficients were calculated. It was assumed that a value of the Cronbach’s alpha coefficient of at least 0.70 indicated the reliability of the scale. A Guttman split-half coefficient value of at least 0.80 was expected to confirm the internal consistency of the scale. Furthermore, item-to-total score correlations were calculated for each item with a relevant function. The expected value of the item-to-total correlation was established at the level of at least 0.40. 

### 2.6. Validity

The adequacy of the samples of respondents in relation to the number of items in the Pl-eHEALS was assessed with the Kaiser-Meyer-Olkin value (expected value > 0.70). The factorability of the data was examined with the Bartlett’s test of sphericity. Apart from an exploratory factor analysis, the hypothesis testing was applied for assessing the validity of the scale. 

Exploratory factor analysis (EFA) was applied to assess the underlying latent variable responsible for the variance of the measure. The extraction of factors was based on the maximum likelihood extraction. In line with the Kaiser criterion, the valid level of the eigenvalue of the extracted factors was set at a level of at least 1. Scree plots were used to visualize the factors to be retained.

The construct validity of the tool was also assessed through hypothesis testing. As the questionnaires used in each survey had a different structure, the hypothesis testing was based on different assumptions. In the survey of the 50+ adult respondents, the relation between their readiness to use the Internet as a primary source of health information and the intensity of their Internet use was determined. For the survey of 18–35 years old women, the association of the level of ehealth literacy with the readiness to use the Internet as a primary source of health information and to undertake health-related activities in the Internet was assessed. The association between ehealth literacy and concrete variables reflecting Internet use and Internet-based health-related activities was assessed by using the non-parametric Mann–Whitney U test.

## 3. Results

### 3.1. Characteristic of the Study Groups

The telephone-based survey targeting adults 50+ (Sample No 1) involved 1000 respondents. The mean age of the respondents was (mean ± SD) 64.16 ± 9.55, of whom 41.5% (*n* = 415) were aged at least 65 with 55.8% (*n* = 558) of the sample being female. According to the main statistical office in Poland (Statistics Poland, SP), the population aged 50 years or more in 2018 was 53.99% female [63]. Of the sample, 48.90% (*n* = 489) were users of the Internet. The mean Pl-eHEALS score was 25.26 ± 5.94, with a median of 25.00 and an interquartile range of 7.00. The mean item score was 3.16 ± 0.74, with item eight achieving the lowest mean score (2.89 ± 0.97) and item five of the eHEALS scale returning the highest score (3.28 ± 0.99). Among respondents who were Internet users, those who used the Internet several times weekly made up 83.84% (*n* = 410), and those who declared that the Internet was, for them, a primary source of health related information comprised 51.53% (*n* = 252).

Sample No 2 consisted of 1030 women aged between 18–35 years old, of whom 41.74% (*n* = 430) were residents of rural areas (according to SP, 42.27% of the women in the age group 20–34 years old lived in rural areas in 2018 [63]), and 19.90% (*n* = 205) resided in urban areas with a population greater than 200,000. The mean total Pl-eHEALS score was (mean ± SD) 29.46 ± 5.05. The range of the means of items included in the Pl-eHEALS was from 3.15 ± 1.03 for item eight to 3.89 ± 0.81 for item two. Using the Internet as a primary source of health-related information was indicated by 50.19% (*n* = 518) of the respondents. Health-related activities performed on the Internet were declared by the following percentages of respondents: asking questions to the health expert, 31.46% (*n* = 324); advice to other Internet users, 20.58% (*n* = 212); receiving advice from other Internet users, 66.70% (*n* = 687); asking questions on health forums, 27.67% (*n* = 285); checking information about ordered medication or tests, 66.12% (*n* = 681); and checking information about physicians or health institutions, 47.48% (*n* = 489). 

### 3.2. Reliability

No significant floor or ceiling effects were observed in Sample No 1 (floor effect 3.00%, ceiling effect 0.2%) or in Sample No 2 (floor effect 0%, the lower level was 10 for 0.10% respondents, ceiling effect 3.1%). The Cronbach’s alpha coefficients were 0.90 for Sample No 1 and 0.88 for Sample No 2, confirming the internal consistency of the Pl-eHEALS scale for both populations. The Guttman split-half coefficients were 0.89 and 0.81, respectively, supporting the internal consistency of the scale. 

The values of Cronbach’s alpha after removing individual items were lower than the combined Cronbach’s alpha except for the item 8 in Sample No 1 (Cronbach’s alpha after removing this item was 0.91) (Table 1). In Sample No 2, all values of Cronbach’s alphas calculated after removing individual items were lower than 0.88 (Table 1). The correlation of individual items to the total score ranged from 0.354 (for item eight) to 0.771 (for item five) in Sample No 1 and from 0.582 (for item eight) to 0.708 (for item five) in Sample No 2 (Table 1).

### 3.3. Validity

#### 3.3.1. Exploratory Factor Analysis

The significant results of the Bartlett’s test of sphericity confirmed the factorability of the correlation matrix in case of both studied populations (sample No 1: chi2 = 2233.48, *p* < 0.001; sample No 2: chi2 = 3641.12, *p* < 0.001). The adequacy of both samples was assured by the results of the Kaiser-Meyer-Olkin test: 0.912 for Sample No 1 and 0.900 for Sample No 2. 

EFA based on the maximum likelihood method revealed that single factor models were valid for both samples. A unidimensional latent structure for both samples could be also seen on the scree plots. Initial eigenvalues were of 4.778 and 4.405, respectively. In the single factor models, the sum of squared loadings of the eight items on the extracted factor using the maximum likelihood method was 6.090 for Sample No 1. It was responsible for 59.72% of the variance in the scale. For Sample No 2, the sum of the squared loadings was 5.927, accounting for 55.06% of the variance. Factor loadings for both samples studied are shown in Table 2.

#### 3.3.2. Hypothesis Testing

The results of the hypothesis testing in both samples demonstrated the construct validity of the Pl-eHEALS. The hypothesis testing undertaken for Sample No 1 revealed that the respondents who used the Internet more frequently (several time a week vs. less frequently, 25.53 ± 5.87 vs. 23.71 ± 6.06, Mann–Whitney U test, Z = −2.16. *p* = 0.031) demonstrated higher levels of health literacy. Furthermore, those who showed a greater readiness to use the Internet for researching health-related information also demonstrated greater ehealth literacy (26.06 ± 5.85 vs. 24.40 ± 5.92, respectively, Mann–Whitney U test, Z = 2.68, *p* = 0.007). 

For Sample No 2, a higher readiness to use the Internet as a primary source of information about health was associated with significantly greater ehealth literacy (30.68 ± 4.67 vs. 28.22 ± 5.12, Mann–Whitney U test, *p* < 0.001). In addition, the respondents undertaking specific health-related activities in the Internet possessed a higher level of ehealth literacy. This association was confirmed for activities including posing questions to a health expert, advising or obtaining advice from other Internet users, asking health-related questions on discussion forums, and checking information about prescribed medication or ordered tests (Table 3). A higher level of ehealth literacy was demonstrated by respondents who checked information about physicians or health institutions on the Internet than by those who did not claim such activity, but the difference was not statistically significant (Table 3).

## 4. Discussion

The study confirmed the reliability and the validity of the Polish version of eHEALS scale in two large different population samples: adults at least 50 years old and women aged 18–35 years. The survey undertaken with adults 50+ was based on the telephone technique, and that on young adult women was carried out online. Less than 50% of respondents in the first sample made use of the Internet. In addition, it should be pointed out that, according to the Eurostat report, the Internet is used by about 99% of young adult women in Poland [64]. Therefore, the technique of CAWI applied for this population was appropriate for ensuring the sample was representative of this segment of Polish society. The Cronbach’s alpha coefficients were 0.90 for Sample No 1 and 0.88 for Sample No 2. The Guttman split-half coefficients in these samples were 0.892 and 0.811, respectively. These results are in agreement with other studies assessing the reliability of the eHEALS instrument. 

The original assessment of the eHEALS carried out by Norman and Skinner [12] yielded a Cronbach’s alpha of 0.88. In other studies published to date, the Cronbach’s alpha ranged from 0.85 for the Serbian [57] to 0.93 for the Japanese [18] and the Dutch versions [15], and 0.95 for the Chinese version of the instrument [40]. Neter et al. reported a Cronbach’s alpha coefficient of 0.86 for the six item version of the eHEALS scale [19]. In the study of Chang et al., the Guttman split-half coefficient was 0.92 [40]. 

To date, the eHEALS scale has been validated in surveys applying three different techniques for questionnaire distribution: paper-and-pencil [15,35,64], telephone-based [15,19,28], and web-based interviewing [18,21,22,26,52,64].

The exploratory factor analysis yielded a single factor structure of the Polish version of the scale. The single factor structure of the original tool was confirmed by Norman and Skinner in a sample of adolescents [12]. It seems that, in most studies in which translated versions of the eHEALS scale were validated, a single factor structure was reported [15,52,55]. A single structure was also reported in other studies using the original English version of the eHEALS [65]. There were also some studies in which a two factor structure was found [19,47], and in one study, a three-dimensional structure was reported [37]. 

In this study, the single factor model explained 59.72% of the variance in the scale for the sample of older adults and 55.06% of variance for the sample of 18–35 years old women. The lowest value (50.3%) of the explained variance thus far reported was for the Korean version of eHEALS validated on a sample of younger adults recruited online [49]. The authors assessing the validity of the Iranian version of the eHEALS reported that maximum likelihood method yielded a single factor solution explaining 70.5% of the variance in the scale [35]. 

The validity of the Pl-eHEALS was additionally checked by hypothesis testing. The analysis confirmed that higher ehealth literacy measured with this instrument in both nationally representative Polish samples was associated with higher respondent’s acceptance of the Internet as a primary source of health-related information. In Sample No 1, higher ehealth literacy was also associated with more frequent use of the Internet and in Sample No 2 was associated with undertaking specific health-related activities online, e.g., asking a health expert or obtaining or providing health-related advice. In those studies in which hypothesis testing was used for the validation of the eHEALS scale, higher ehealth literacy was shown to be related to more intensive Internet use [15], years of experience using the Internet [26], hours per week of Internet use [29], computer knowledge [26], computer and technology skills [40], and computer literacy [35]. In some studies, non-Internet related variables were used for hypothesis testing, e.g., indicators of psychological wellbeing [52,55] and lifestyle habits [55].

The Polish validated version of eHEALS provided another tool to facilitate the assessment of ehealth literacy in Poland. For many years, the country was lagging behind other states in the development of the information society. Poland’s accession to the European Union in 2004 provided a great boost to the growth of the country’s information infrastructure. The rapid growth of Internet use is also evident in the context of health related applications. However, for many years, no standardized tool was available to measure the ehealth-related competencies of Polish society. The introduction of the Pl-eHEALS has enabled research to be conducted in this domain and, using standardized approaches to measuring ehealth literacy, the results compared with those of other countries.

### Limitations

The process of transcultural adaptation was carried out with the inclusion of only one forward and one backward translator. This could limit the perception of possible phrasing of specific items. Furthermore, the piloting of the questionnaire was performed in a group of public health students that generally demonstrate more accepting attitudes to the Internet use for health-related purposes. 

To overcome any bias resulting from testing a new instrument on a specific population, the assessment of the validity and the reliability was carried out using two nationally representative samples having significantly different ages. However, the design of the survey did not allow for test-retest assessment of the reliability; therefore, the Guttman split-half coefficient was calculated. For the survey performed as a CATI, there is the possibility that the selection was biased in favor of respondents who remained at home. Therefore, the 50+ group of respondents could represent more uniform sample in terms of social and professional activity. However, this in turn could result in its lower representativeness, especially when we consider the potentially professionally active population 50–65 years old.

## 5. Conclusions

The Polish version of the eHEALS instrument was developed according to the guidelines of transcultural adaptation. The tool demonstrated good reliability and validity in both surveys carried out using different survey techniques. EFA revealed a single factor model typically found for the eHEALS questionnaire and confirmed in most other studies.

## Figures and Tables

**Table 1:** Means, item-to-total correlations, and Cronbach’s alphas after removing specific items of the Polish version of the ehealth literacy scale (Pl-eHEALS) for both samples.

Item	Sample No 1				Sample No 2			
	Mean after Removing Item	Variance of the Scale after Removing Item	Item-to-Total Correlation	Cronbach’s Alpha after Removing Item	Mean after Removing Item	Variance of the Scale after Removing Item	Item-to-Total Correlation	Cronbach’s Alpha after Removing Item
item 1	22.12	28.131	0.659	0.887	25.79	20.461	0.613	0.868
item 2	22.05	26.502	0.764	0.877	25.75	19.959	0.671	0.862
item 3	22.03	26.417	0.767	0.877	25.68	19.767	0.691	0.860
item 4	22.01	27.039	0.745	0.879	25.62	20.034	0.643	0.865
item 5	21.98	26.477	0.771	0.876	25.69	19.448	0.708	0.858
item 6	22.11	26.554	0.761	0.877	25.88	19.337	0.657	0.863
item 7	22.12	27.543	0.648	0.888	25.85	19.687	0.618	0.867
item 8	22.37	30.533	0.354	0.914	26.35	19.046	0.582	0.874

**Table 2:** Factor loadings and factor score coefficients for the Pl-eHEALS and single extracted factor for Sample No 1 (*n* = 489) and Sample No 2 (*n* = 1030).

Item	Factor Loadings * Sample No 1	Factor Loadings * Sample No 2
item 1	0.739	0.712
item 2	0.842	0.769
item 3	0.850	0.786
item 4	0.826	0.744
item 5	0.847	0.794
item 6	0.827	0.738
item 7	0.731	0.708
item 8	0.429	0.676
Sum of squared loadings	6.090	4.778

* The single factor was extracted using the maximum likelihood method.

**Table 3:** The association of ehealth literacy and health-related activities performed online (Sample No 2).

Variable	*n*	eHEALS Mean ± SD	*p* *
Questions to health expert in the Internet			
no	706	28.95 ± 5.00	<0.001
yes	324	30.56 ± 4.99	
Advising other Internet users about health issues			
no	818	29.17 ± 5.05	<0.001
yes	212	30.58 ± 4.88	
Obtaining advice about health from other Internet users			
no	343	28.94 ± 5.50	0.043
yes	687	29.71 ± 4.79	
Asking questions about specific health issues on discussion fora			
no	745	29.18 ± 5.13	0.005
yes	285	30.19 ± 4.75	
Checking the information about prescribed medications or ordered tests			
no	365	28.90 ± 5.45	0.037
yes	665	29.76 ± 4.79	
Checking the information about physicians or health institutions			
no	349	29.19 ± 5.23	0.15
yes	681	29.60 ± 4.95	

* - *p* for the Mann–Whitney U test.
